# Predictive Performance of a Gentamicin Pharmacokinetic Model in Term Neonates with Perinatal Asphyxia Undergoing Controlled Therapeutic Hypothermia

**DOI:** 10.1097/FTD.0000000000001166

**Published:** 2024-01-24

**Authors:** Marlotte A. A. van der Veer, Timo R. de Haan, Linda G. W. Franken, Floris Groenendaal, Peter H. Dijk, Willem P. de Boode, Sinno Simons, Koen P. Dijkman, Henrica L.M. van Straaten, Monique Rijken, Filip Cools, Debbie H. G. M. Nuytemans, Anton H. van Kaam, Yuma. A. Bijleveld, Ron A. A. Mathôt, Mieke J. Brouwer, Marcel P. van den Broek, Carin M. A. Rademaker, Djien Liem, Katerina Steiner, Annelies A. Bos, S. M. Mulder-de Tollenaer, L. J. M. Groot Jebbink-Akkerman, Michel Sonnaert, Fleur Anne Camfferman

**Affiliations:** * Department of Pharmacy & Clinical Pharmacology, Amsterdam University Medical Center, Amsterdam, the Netherlands;; † Department of Neonatology, Emma Children's Hospital, Amsterdam University Medical Center, Amsterdam, the Netherlands;; ‡ Department of Neonatology, Wilhelmina Children's Hospital, Utrecht, The Netherlands;; § UMC Utrecht Brain Center, University Medical Center Utrecht and Utrecht University, Utrecht, The Netherlands;; ¶ Division of Neonatology, Department of Pediatrics, University Medical Center Groningen, Beatrix Children's Hospital, University of Groningen, Groningen, the Netherlands;; ║ Department of Neonatology, Radboud University Medical Center, Radboud Institute for Health Sciences, Amalia Children's Hospital, Nijmegen, The Netherlands;; ** Department of Neonatal and Pediatric Intensive Care, Division of Neonatology, Erasmus MC-Sophia Children's Hospital, Rotterdam, The Netherlands;; †† Department of Neonatology, Máxima Medical Center Veldhoven, Veldhoven, The Netherlands;; ‡‡ Department of Neonatology, Isala Clinics, Zwolle, The Netherlands;; §§ Department of Neonatology, Willem-Alexander Children's Hospital, Leiden University Medical Center, Leiden, The Netherlands; and; ¶¶ Department of Neonatology, Vrije Universiteit Brussel, Brussels, Belgium.

**Keywords:** asphyxia, external validation, gentamicin, neonates, therapeutic hypothermia

## Abstract

**Background:**

Model validation procedures are crucial when population pharmacokinetic (PK) models are used to develop dosing algorithms and to perform model-informed precision dosing. We have previously published a population PK model describing the PK of gentamicin in term neonates with perinatal asphyxia during controlled therapeutic hypothermia (TH), which showed altered gentamicin clearance during the hypothermic phase dependent on gestational age and weight. In this study, the predictive performance and generalizability of this model were assessed using an independent data set of neonates with perinatal asphyxia undergoing controlled TH.

**Methods:**

The external data set contained a subset of neonates included in the prospective observational multicenter PharmaCool Study. Predictive performance was assessed by visually inspecting observed-versus-predicted concentration plots and calculating bias and precision. In addition, simulation-based diagnostics, model refitting, and bootstrap analyses were performed.

**Results:**

The external data set included 323 gentamicin concentrations of 39 neonates. Both the model-building and external data set included neonates from multiple centers. The original gentamicin PK model predicted the observed gentamicin concentrations with adequate accuracy and precision during all phases of controlled TH. Model appropriateness was confirmed with prediction-corrected visual predictive checks and normalized prediction distribution error analyses. Model refitting to the merged data set (n = 86 neonates with 935 samples) showed accurate estimation of PK parameters.

**Conclusions:**

The results of this external validation study justify the generalizability of the gentamicin dosing recommendations made in the original study for neonates with perinatal asphyxia undergoing controlled TH (5 mg/kg every 36 or 24 h with gestational age 36–41 and 42 wk, respectively) and its applicability in model-informed precision dosing.

## Abstract 2

Supplemental Digital Content is Available in the Text.

## BACKGROUND

Hypoxic–ischemic encephalopathy resulting from perinatal asphyxia is a severe clinical condition with significant morbidity and mortality rates among term neonates.^1^ Controlled therapeutic hypothermia (TH) is the current standard of care for neonates with moderate-to-severe hypoxic–ischemic encephalopathy after perinatal asphyxia.^2^ Its objective is to enhance long-term neurodevelopmental outcomes by reducing and stabilizing core body temperature to 33.5°C for 72 within 6 hours of the hypoxic–ischemic event, followed by gradual rewarming to normothermia (36.5°C).^3^

In the Netherlands and Belgium, the aminoglycoside gentamicin is frequently prescribed as part of the first-line therapy for neonates with perinatal asphyxia because early-onset bacterial sepsis cannot be reliably ruled out.^4^ Gentamicin exhibits a narrow therapeutic index and substantial interindividual variability in its pharmacokinetics (PK), with a heightened risk of nephrotoxicity and ototoxicity associated with elevated trough levels.^5^ Model-informed precision dosing (MIPD) of gentamicin is recommended to personalize dosing and attain appropriate peak and trough levels.^6^ However, dosing gentamicin in neonates undergoing controlled TH is challenging because gentamicin clearance is altered during hypothermia, increasing the risk of toxicity.^7^ Our group previously developed a gentamicin PK model that accounts for this altered clearance and interindividual variability, which can enhance MIPD performance.^8^

Validating population PK models is crucial when using them to develop dosing algorithms and perform MIPD. Ideally, the external generalizability of a population PK model is evaluated using independent data.^9^ However, there is a scarcity of external validation studies, especially in neonatal populations, because of the limited number of available studies and consequently, external and independent data sets.^10^ In neonates with perinatal asphyxia undergoing controlled TH, only 2 population PK models for antibiotics (amoxicillin and gentamicin) have been partially externally validated.^11,12^

Among the 3 published gentamicin PK models in this specific patient population, our study presented the first PK model that incorporated prospectively collected data sampled at multiple time points throughout all phases of controlled TH.^8,13,14^ This model was validated using advanced internal methods [bootstrap and normalized prediction distribution error (NPDE) analysis], but no external validation had been conducted.^8^ In this study, we assessed the predictive performance and generalizability of our original gentamicin PK model using an independent data set of (near) term neonates with perinatal asphyxia undergoing controlled TH.

## MATERIALS AND METHODS

### Study Design and Population

Data for our original PK model (model-building data set) and for the external evaluation of this model (external dataset) were both sourced from the PharmaCool Study, a comprehensive multicenter prospective observational cohort study conducted across all 10 Dutch and 2 Belgian Neonatal Intensive Care Units (https://trialsearch.who.int, NTR2529).^8^ Detailed information on the study design is available elsewhere.^3^ The external validation data set did not contain patient data that had already been used in the development of the original PK model.

In brief, neonates of (near) term gestational age (GA >36 weeks) with perinatal asphyxia were enrolled if they met the criteria for controlled TH.^3^ Exclusion criteria included the presence of congenital hepatic or renal pathology, lack of central venous line or arterial bloodstream access for sample collection, or absence of parental consent.

### Data and Sample Collection

Patient-specific, demographic, clinical, and laboratory data were systematically collected for each patient, encompassing GA, birthweight, sex, Thompson score, Apgar score, cause of asphyxia, extent and duration of resuscitation, requirement for ventilator and/or inotropic support, comedication, mean daily urinary output, serum creatinine, urea, aspartate aminotransferase, alanine aminotransferase, and the presence of multiorgan failure.^3^ Gentamicin was administered to each patient through 30-minute infusions of 4 mg/kg every 24 hours^15^ Detailed dosing and sample collection schedules were recorded in a digital case report form. In the clinical context, concentration-guided dosing adhered to local protocols established by individual centers.

Blood samples were collected from indwelling arterial lines at specified time points during hypothermia (day 2 and 3), rewarming (day 4), and normothermia (day 5).^3^ After collection, blood samples were transported to the hospital pharmacy laboratory of the Amsterdam University Medical Centre, stored at −80°C, and analyzed using a validated liquid chromatography mass spectrometry method, as previously described.^16^

### Original Model

The development and validation of the original gentamicin PK model was conducted using a first-order conditional estimation with interaction algorithm in the nonlinear mixed effects modeling software NONMEM (version 7.4.2; ICON Development Solution, Gaithersburg, MD). The final model comprised an allometrically scaled two-compartment model with GA as a covariate on clearance. Gentamicin clearance remained constant during hypothermia and rewarming but increased on study day 5, when normothermia was reached. A categorical covariate on clearance was introduced, taking a value of 0 before study day 5 and 1 on study day 5.^8^

Based on simulations, a model-based empiric dosing algorithm of 5 mg/kg gentamicin every 36 hours or every 24 hours was recommended for neonates with GA of 36–41 weeks and 42 weeks, respectively.^8^ The dosing regimens proposed in this study were subsequently adopted by the Dutch Paediatric Formulary.^15^

### External Model Validation and Evaluation

For each neonate in the external data set, model-based population-predicted concentrations were computed by locking the final parameters of the original model through the NONMEM MAXEVAL = 0 POSTHOC command.^17^ These population-predicted concentrations were graphically compared with the corresponding observed concentrations for all levels, as well as separately for low and high concentration levels. This comparative analysis was conducted across all phases of controlled TH and during both the hypothermic and normothermic phases independently. The predictive performance was assessed using bias and precision, which were calculated using the following equations^18^:

Prediction error (PE)=predicted−observed

$$\text{Bias}:\text{Mean}\;\text{prediction}\;\text{error}\;\left(\text{MPE}\right)=\frac{\overset{N}{\underset{j=1}{\sum }}\;\left({\text{PE}}_{j}\right)}{N}$$

$$\text{Precision}:\text{Root}\;\text{mean}\;\text{squared}\;\text{error}\;\left(\text{RMSE}\right)=\sqrt{\frac{\overset{N}{\underset{j=1}{\sum }}\;{\left({\text{PE}}_{j}\right)}^{2}}{N}}$$

where *predicted* refers to the model-predicted gentamicin concentrations, *observed* pertains to the measured gentamicin concentrations, and *N* represents the number of pairs. To comprehensively assess the predictive performance of the model across different phases of controlled TH, bias and precision were computed for gentamicin concentrations during both the hypothermic and normothermic phases. In addition, a distinction was made between low and high gentamicin levels because a higher bias or lower precision would have more significant implications for low gentamicin concentrations (typically trough levels) compared with high concentrations (typically peak levels).

Because gentamicin was often discontinued after a single dose, and blood samples were collected at fixed intervals regardless of dosing times, there were a limited number of true trough levels available for analysis. Consequently, rather than relying solely on trough levels, a cutoff of a gentamicin concentration of ≤1.5 mg/L was established. This approach allowed for a direct comparison between the actually measured low gentamicin concentrations and the population-predicted concentrations derived from the original PK model. High gentamicin levels were defined as samples taken within 2 hours after the preceding administered dose because all the highest gentamicin concentrations were measured within this time interval, aligning with a previous study's approach.^19^

To further evaluate predictive performance, a prediction-corrected visual predictive check (pcVPC) and a NPDE analysis were conducted, both with n = 1000 simulations.^20,21^ Subsequently, if no apparent trends, imprecision, or bias were detected in the previous steps, the model building and external data sets were merged and jointly analyzed by refitting the merged data set to the original gentamicin PK model. A parameter obtained from the model refit was deemed accurate if it deviated by less than 20% from the original model fit. A covariate analysis on the merged data set was performed once again, using a forward and backward selection process. A decrease in the objective function value of ≥3.8 points was considered statistically significant in the first step, followed by a more stringent decrease in objective function value of ≥10.83 (*P*-value of <0.001) in the second part. Finally, a pcVPC of the merged data set was generated, and the robustness of the refitted model was assessed through a bootstrap analysis.

## RESULTS

The external data set comprised 39 neonates, providing a total of 323 gentamicin samples for analysis. Table 1 presents the baseline characteristics of neonates from both the model-building data set and the external data set. Notably, neonates in the external data set received gentamicin treatment for a shorter duration, resulting in a reduced number of available plasma samples per patient. Most of the plasma samples (80%) in the external data set were collected during the hypothermic phase. Furthermore, most neonates in the external data set received treatment at different centers compared with those in the model-building data set. However, other characteristics were similar between the 2 data sets.

**TABLE 1.** Patient Characteristics and Samples Drawn

Characteristic*	Model-Building Data Set	External Data Set
Subjects (n)	47	39
Male, n (%)	27 (58.7)	22 (56.4)
Birth weight, grams	3400 (2090–5070)	3170 (2260–4620)
GA, wks†	40 (36–42)	40 (36–42)
PNA, d‡	4.7 (2.3–5.2)	3.1 (1.5–4.9)
SCr (µmol/L)§	49 (26–114)	73 (29–167)
Urine output (mL/kg/h)	3.0 (0.1–7.6)	2.5 (0.01–7.1)
Thompson score†	9 (3–19)	9 (6–15)
MOF, n (%)§	19 (40.4%)	13 (33.3%)
Duration gentamicin treatment, d¶	2 (0–5)	1.5 (0–4)
Daily gentamicin dose (mg/kg)	4.0 (3.5–5.1)	4.0 (3.0–5.9)
Total number of samples		
During study period	612	323
Per patient during study period*	14 (4–16)	8 (2–15)
During hypothermic phase (%)	386 (63)	258 (80)
Per patient during hypothermic phase*	9 (3–9)	7 (2–9)
During normothermic phase (%)	219 (36)	65 (20)
Per patient during normothermic phase*	6 (0–6)	1 (0–6)

* Baseline characteristics are depicted by median and range for continuous variables and percentages for categorical variables.

† Measured at admittance.

‡ Measured at the end of the study period.

§ Measured throughout the study period.

¶ Gentamicin treatment duration of 0 days indicates that a single dose was administered.

MOF, multiorgan failure; PNA, postnatal age; SCr, serum creatinine.

**FIGURE 1.**
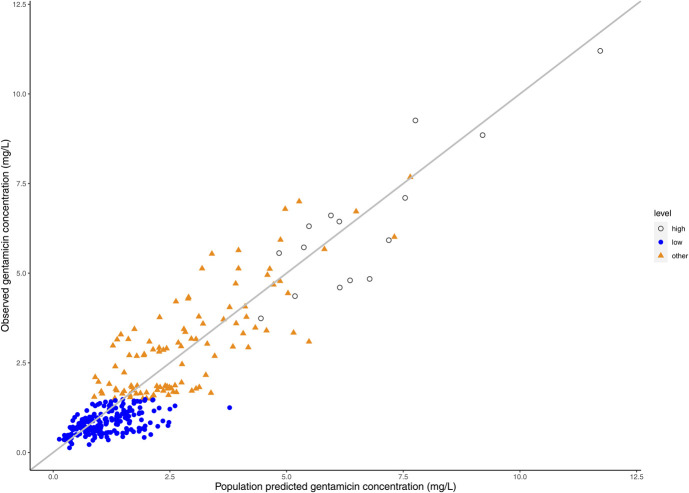
Predicted-versus-observed plots depicting gentamicin concentrations for all phases, including low levels (≤1.5 mg/L) and high levels (<2 hours after the previous dose) during controlled TH. Separated plots for the hypothermic and normothermic phases are provided in **Supplemental Digital Content 1** (see **Figure**, http://links.lww.com/TDM/A705).

**FIGURE 2.**
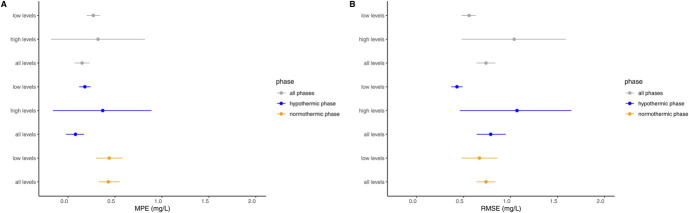
Gentamicin pharmacokinetic model prediction error during all phases of controlled TH, encompassing the hypothermic, normothermic, and combined phases. Prediction error is assessed through bias, represented by (A) mean prediction error in mg/L, and precision, illustrated by (B) RMSE in mg/L. Closed circles denote the mean values with accompanying 95% confidence intervals. Low levels indicate gentamicin concentrations ≤1.5 mg/L, and high levels represent gentamicin concentrations between 0 and 2 hours after dosing. Prediction error for high levels during the normothermic phase is not presented because of the limited number of samples during this phase.

Figure 1 illustrates the predicted-versus-observed plot for all phases of controlled TH, while **Supplemental Digital Content 1** (see **Figure**, http://links.lww.com/TDM/A705) displays the predicted-versus-observed plots for the hypothermic and normothermic phases separately. Bias and precision for all concentration levels, both high and low, during the various phases are presented in Figure 2 and **Supplemental Digital Content 1** (see **Table**, http://links.lww.com/TDM/A705). Notably, no discernible trends were observed in the predicted-versus-observed plots or in the bias and precision calculations for all concentrations during all phases of controlled TH. Some slight overprediction was noted when focusing on low levels during the normothermic phase, meaning the model tended to predict slightly higher values than measured. However, no such overprediction was observed during the hypothermic phase, and precision was generally higher for low levels compared with high levels.

**FIGURE 3.**
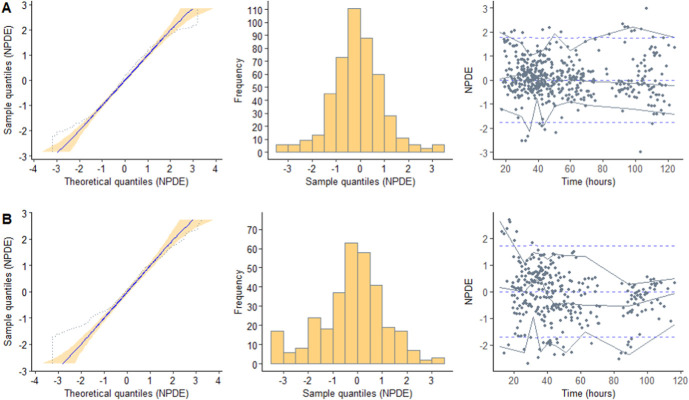
Results obtained from the NPDE analysis, using (A) the model-building data set and (B) the external data set. The NPDE distribution displays a mean and variance of −0.0965 and 1.156, respectively, for the model-building data set, and −0.2087* and 1.726*, respectively, for the external data set (* indicates a statistically significant difference from 0 for mean and 1 for variance (*P* < 0.05) as determined by the Wilcoxon signed-rank test and Fisher test of variance). The TH period encompasses the hypothermic phase (0–72 hours), rewarming phase (72–96 hours), and normothermic phase (>96 hours).

**FIGURE 4.**
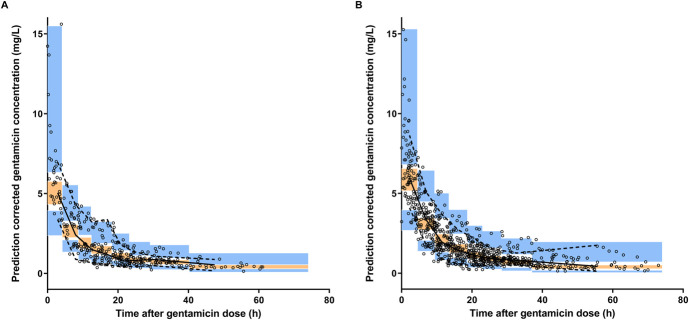
Prediction-corrected visual predictive checks (pcVPC) for (A) the external data set using the previously published gentamicin model and (B) the merged data set, which includes both the model-building and external data sets. The black open circles represent observed gentamicin concentrations, while the black solid line denotes the observed median, and the black dashed lines depict the 5th and 95th percentiles. The orange area signifies the 95% CI of the model-predicted median, and the blue area indicates the model-predicted 5th and 95th percentiles.

The NPDE analysis revealed a mean of −0.2087 and a variance of 1.726, both significantly different from the expected mean of 0 and variance of 1 (as indicated by the Wilcoxon signed-rank test and Fisher test of variance, *P* < 0.05) for the external data set. This suggests that the original covariate model overpredicted the variability of the external data set (Fig. 3). Importantly, no bias became apparent when plotting the NPDE against time after the dose. In addition, the pcVPC of the external data set demonstrated a good overlay between the median, fifth, and 95th percentiles of the simulations from the original model and the observations from the external data set. This indicates that the population PK of gentamicin in the external data set is adequately described (Fig. 4).

Table 2 provides an overview of the parameter estimates for the model-building data set, the merged data set, and the bootstrap analysis. Notably, none of the parameters obtained from the model refit using the merged data set deviated from the original PK model. As depicted in the goodness-of-fit plots in **Supplemental Digital Content 1** (see **Figure**, http://links.lww.com/TDM/A705), the refitted model using the merged data set exhibited an acceptable fit. Furthermore, no new covariate relationships could be identified in the merged data set. These results from external validation reaffirm the generalizability of the dosing regimen of 5 mg/kg gentamicin every 36 hours or every 24 hours for neonates with GA of 36–41 and 42 weeks, respectively.

**TABLE 2.** Parameter Estimates of the Model-Building Data Set and the Refit and Bootstrap of the Merged Data Set

Parameter	Model-Building Data Set [Estimate (CV%)]	Refit: Merged Data Set [Estimate (CV%)]	Bootstrap: Merged Data Set		
			Estimate	CI (2.5%)	CI (97.5%)
CL (L/h/70 kg)	1.89 (5)	1.88 (3)	1.87	1.77	2.00
V_c_ (L/70 kg)	32.5 (10)	33.2 (6)	33.2	29.0	38.0
Q (L/h/70 kg)	2.01 (12)	1.81 (7)	1.83	1.42	2.21
V_p_ (L70 kg)	30.3 (9)	32.2 (8)	32.3	27.3	38.9
Additive error	0.15 (9)	0.16 (7)	0.16	0.14	0.19
IIV on CL (%)	26.6 (15)	24.5 (12)	23.7	17.9	29.4
IIV on V_c_ (%)	40.8 (22)	30.1 (24)	29.8	14.1	41.8
IIV on V_p_ (%)	53.3 (23)	56.4 (20)	55.7	32.7	84.5
IIV on additive residual error (%)	50.2 (15)	52.3 (12)	51.5	36.9	63.7
Θ_CLGA_	3.0 (16)	2.76 (15)	2.63	1.35	3.77
Θ_SD5_	1.29 (12)	1.36 (4)	1.36	1.14	1.52

Final model: TVCL = CL × (BW/70)^0.75^ × θ_SD5_ × (GA/GA_median_)^θCLGA^; TVV_c_ = V_c_ × (BW/70)^1^; TVQ = Q × (BW/70)^0.75^; TVV_p_ = V_p_ × (BW/70).^1^

CL, clearance; Θ_CLGA_, fractional change in CL with each unit of deviation from the median GA; CV, coefficient of variation; IIV, interindividual variability; Θ_SD5_, study day 5 (>96 h postnatal age); Q, intercompartmental clearance; V_c_, volume of distribution of the central compartment; V_p_, volume of distribution of the peripheral compartment.

## DISCUSSION

In this study, we conducted an external validation of a previously published gentamicin population PK model using a cohort of comparable (near) term neonates with perinatal asphyxia undergoing controlled TH. While several population PK models for gentamicin in cooled neonates exist, our model stands out as the first to undergo comprehensive external validation.

Validation and refinement of population PK models are critical steps when considering their application in clinical practice, especially when developing new dosing regimens and implementing precision dosing in vulnerable populations like neonates with perinatal asphyxia undergoing controlled TH. These populations often present challenges such as limited sample sizes and high interpatient variability. Unfortunately, external validations of PK models are infrequently conducted, with only 7% of published models undergoing such scrutiny.^22^ A recent systematic review of antibiotic PK models revealed that only 37 models had undergone thorough external validation, with more than half of them being vancomycin PK models. Moreover, among the 5 pediatric PK models that were externally validated, none were validated in neonates.^23^ This scarcity of external validation studies can be attributed in part to the absence of guidelines for population PK modeling, which hampers the precise and reliable utilization of PK models in clinical settings.^24^

In this study, we overcame these challenges by using an independent data set and using multiple validation tools to evaluate the predictive performance of our previously developed gentamicin PK model in neonates with perinatal asphyxia undergoing controlled TH. Our findings demonstrate that the model performed well in predicting gentamicin concentrations during all phases of controlled TH. Overall, the model exhibited a slight tendency to overpredict gentamicin concentrations (MPE: 0.15 mg/L; 95% confidence interval [CI], 0.07–0.23), primarily driven by overpredictions during the normothermic phase (MPE: 0.43 mg/L; 95% CI, 0.33–0.55). Importantly, there was no overprediction during the hypothermic phase (MPE: 0.08 mg/L; 95% CI, −0.02 to 0.17), which is reassuring because most of the samples (80%) were collected during this phase. Moreover, considering the risks associated with high trough gentamicin levels, a slight overprediction may be preferable from a safety standpoint, especially given that renal insufficiency is a recognized complication in (near) term neonates with perinatal asphyxia.^25^

Notably, precision was lower for high gentamicin levels (RMSE: 1.07 mg/L; 95% CI, 0.46–1.65) than for low levels (RMSE: 0.43 mg/L; 95% CI, 0.37–0.49) during the hypothermic phase. This disparity in precision is expected given the inherently greater magnitude of high gentamicin levels compared with low levels. In clinical practice, allowable error margins are typically set at approximately 0.5 mg/L for trough levels, which are generally indicative of low gentamicin concentrations, and 2 mg/L for peak levels.^26^ However, it is worth noting that imprecision when estimating a priori trough levels using gentamicin models in neonates is not uncommon.^26^ In a recent study that compared model-to-model predictions using 6 previously published neonatal gentamicin models, 4 of these models performed reasonably well a priori of bias and precision. The RMSE values for these 4 models ranged from approximately 0.6 to 1.0 mg/L for trough levels, which is somewhat higher compared with our findings.

Through comprehensive simulation-based diagnostic tests, we were able to confirm that the original model accurately predicted gentamicin concentrations, as evidenced by the pcVPC. Despite a slight overestimation of variability in the NPDE analysis, the distribution generally conformed to normal expectations. Given that most neonates in the external data set were treated at different centers compared with the model-building data set, some degree of variability was anticipated. In addition, the neonates in the external data set received gentamicin for a shorter duration, suggesting potential differences in disease states or clinical practices. Nonetheless, after reapplying the model to the merged data sets (encompassing both the model-building data set and the external data set) and subjecting it to a bootstrap analysis, the original PK model for gentamicin in neonates with perinatal asphyxia undergoing controlled TH was deemed robust, with accurately estimated parameters.

These results from the external validation further substantiate the identified covariate associations, reinforcing our confidence in the gentamicin dosing regimens during controlled TH as derived from the original PK model. This original model accounts for variations in GA, temperature (hypothermia), and weight, ensuring its applicability in clinical practice.

It is essential to acknowledge certain limitations in our study. First, the PK model exhibited some degree of lower precision that could not be entirely explained by the inclusion of additional covariates. Nevertheless, the RMSE remained within clinically acceptable bounds. Second, our focus on true trough levels was limited because of their restricted availability. As a result, we opted to categorize gentamicin levels into low and high concentrations, with levels above 1.5 mg/L being excluded. However, a comprehensive examination of all gentamicin levels revealed low bias and high precision. Third, most gentamicin samples in the external data set were collected during the hypothermic phase, thereby primarily assessing the predictive performance of the PK model during the hypothermic phase. Nevertheless, the population PK model consistently demonstrated stability across all phases of controlled TH. Our next step should involve prospective validation of the suitability of the model-derived dosing regimens.

## CONCLUSIONS

This study marks a significant milestone as the first to evaluate the predictive performance of a previously published gentamicin PK model in an independent cohort of (near) term neonates with perinatal asphyxia undergoing controlled TH. The outcomes of this external validation lend robust support to the gentamicin dosing recommendations established in the original study and affirm the model's suitability for MIPD.

## Supplementary Material
