# Supplementary material for: Predictive Performance of a Gentamicin Pharmacokinetic Model in Term Neonates with Perinatal Asphyxia Undergoing Controlled Therapeutic Hypothermia
Source: Ther Drug Monit. 2024 Jan 24;46(3):376–83. doi: 10.1097/FTD.0000000000001166 (PMC11078285; doi:10.1097/FTD.0000000000001166)
Supplement: Supplementary file 1 [file tdm-46-376-s001.docx]

**Supplemental Digital Content**


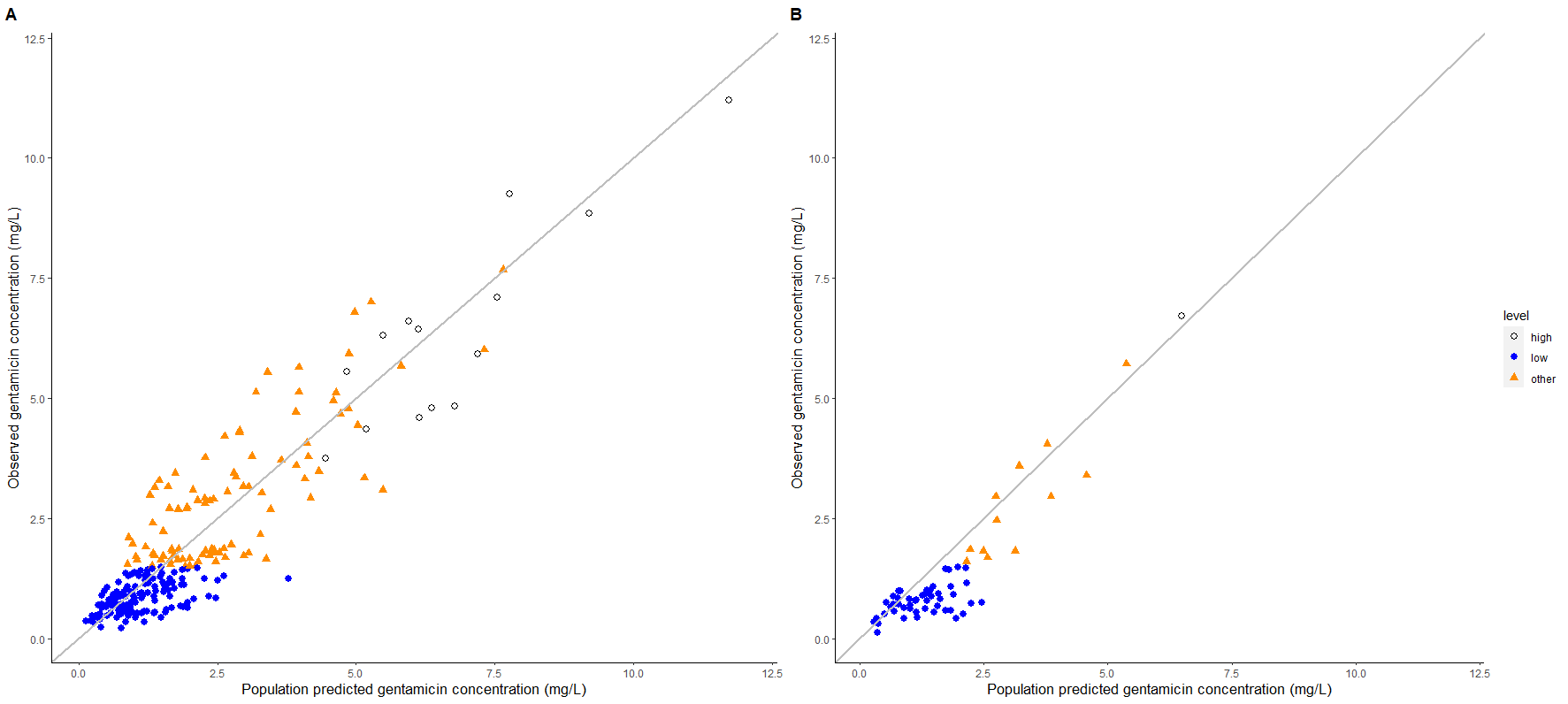
S1: Predicted-versus-observed plots during hypothermia and normothermia

**Figure S1: Results for the predicted-versus-observed plots for low levels (≤ 1.5 mg/L), high levels (< 2 h after previous dose) and other levels during (A) the hypothermic phase and (B) the normothermic phase.**

S2: Calculated values for bias and precision

**Table S1:** Prediction error of the gentamicin pharmacokinetic model during all phases (hypothermic, normothermic, and all phases combined) of controlled therapeutic hypothermia.

| **Level^a^** | **MPE (mg/L)^b^** | **95% CI** | | | **Phase** |
| --- | --- | --- | --- | --- | --- |
| All | 0.15 | 0.07 | 0.23 | All | |
| High | 0.32 | –0.18 | 0.82 | All | |
| Low | 0.27 | 0.2 | 0.34 | All | |
| All | 0.43 | 0.33 | 0.55 | Normothermia | |
| Low | 0.44 | 0.3 | 0.58 | Normothermia | |
| All | 0.08 | –0.02 | 0.17 | Hypothermia | |
| High | 0.37 | –0.16 | 0.89 | Hypothermia | |
| Low | 0.18 | 0.12 | 0.24 | Hypothermia | |

| **Level^a^** | **RMSE (mg/L)^b^** | **95% CI** | | **Phase** |
| --- | --- | --- | --- | --- |
| All | 0.74 | 0.64 | 0.84 | All |
| High | 1.04 | 0.48 | 1.59 | All |
| Low | 0.56 | 0.48 | 0.63 | All |
| All | 0.74 | 0.64 | 0.84 | Normothermia |
| Low | 0.67 | 0.48 | 0.86 | Normothermia |
| All | 0.79 | 0.64 | 0.95 | Hypothermia |
| High | 1.07 | 0.46 | 1.65 | Hypothermia |
| Low | 0.43 | 0.37 | 0.49 | Hypothermia |

^a^ Low levels: gentamicin concentration ≤ 1.5 mg/L and high levels: gentamicin concentration between 0 and 2 h after dose. Prediction errors for high levels during the normothermic phase are not provided as only one sample was measured during this phase. Abbreviations: MPE, mean prediction error; RMSE, root mean square error; ^b^ Prediction error assessed as bias by the MPE in mg/L and as precision by the RMSE in mg/L.

S3: Goodness-of-fit plots


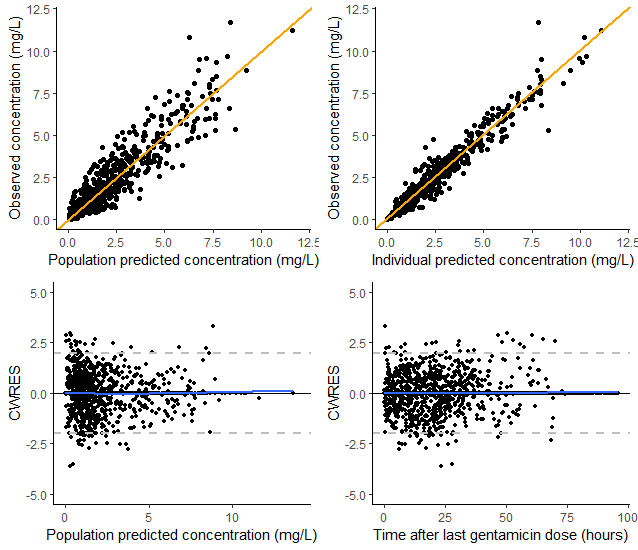


**Figure S2.** Model evaluation of the merged gentamicin dataset (including both the model building and external datasets). Basic goodness-of-fit plots of the refitted population pharmacokinetic model.
